# A multi-national comparison of antipsychotic drug use in children and adolescents, 2005–2012

**DOI:** 10.1186/s13034-017-0192-1

**Published:** 2017-10-11

**Authors:** Luuk J. Kalverdijk, Christian J. Bachmann, Lise Aagaard, Mehmet Burcu, Gerd Glaeske, Falk Hoffmann, Irene Petersen, Catharina C. M. Schuiling-Veninga, Linda P. Wijlaars, Julie M. Zito

**Affiliations:** 1 Department of Psychiatry, University of Groningen, University Medical Center Groningen, Groningen, The Netherlands; 2 Freelance Researcher, Marburg, Germany; 3 Life Science Team, IP & Technology, Bech-Bruun Law Firm, Copenhagen, Denmark; 4 Department of Pharmaceutical Health Services Research, University of Maryland, Baltimore, MD USA; 5 Division of Health Long-term Care and Pensions, University of Bremen, SOCIUM Research Center on Inequality and Social Policy, Bremen, Germany; 6 Department of Health Services Research, Carl von Ossietzky University, Oldenburg, Germany; 7 Department of Primary Care and Population Health, University College London, London, UK; 8 University of Groningen, Pharmacotherapy, Epidemiology & Economics, Groningen, The Netherlands; 9 Population, Policy and Practice, University College London Great Ormond Street Institute of Child Health, London, UK

**Keywords:** Adolescents, Children, Antipsychotic drugs, Atypical, Denmark, Germany, Netherlands, UK, USA, Pharmacoepidemiology

## Abstract

Over the last decades, an increase in antipsychotic (AP) prescribing and a shift from first-generation antipsychotics (FGA) to second-generation antipsychotics (SGA) among youth have been reported. However, most AP prescriptions for youth are off-label, and there are worrying long-term safety data in youth. The objective of this study was to assess multinational trends in AP use among children and adolescents. A repeated cross-sectional design was applied to cohorts from varied sources from Denmark, Germany, the Netherlands, the United Kingdom (UK) and the United States (US) for calendar years 2005/2006–2012. The annual prevalence of AP use was assessed, stratified by age group, sex and subclass (FGA/SGA). The prevalence of AP use increased from 0.78 to 1.03% in the Netherlands’ data, from 0.26 to 0.48% in the Danish cohort, from 0.23 to 0.32% in the German cohort, and from 0.1 to 0.14% in the UK cohort. In the US cohort, AP use decreased from 0.94 to 0.79%. In the US cohort, nearly all ATP dispensings were for SGA, while among the European cohorts the proportion of SGA dispensings grew to nearly 75% of all AP dispensings. With the exception of the Netherlands, AP use prevalence was highest in 15–19 year-olds. So, from 2005/6 to 2012, AP use prevalence increased in all youth cohorts from European countries and decreased in the US cohort. SGA were favoured in all countries’ cohorts.

## Introduction

During the past decades, antipsychotic drugs (AP) have gained popularity as a treatment for psychiatric disorders in young people in most developed countries [1]. AP can be divided in two groups: first generation (typical) antipsychotics (FGA) and second-generation (atypical) antipsychotics (SGA) [2, 3]. Efficacy of AP in youth has been demonstrated for psychotic symptoms [4], bipolar disorder [5], irritability in autistic children [6], tics [7], and some forms of (severe) aggressive behaviour [8, 9]. Ample use of AP drugs has been described in children with a mental handicap and behavioral symptoms [10]. But only few antipsychotic drugs are licensed for those indications and for children and there is a lack of long-term efficacy and safety data [11]. Therefore, the treatment of youth with antipsychotics is subject to debate among clinicians, scientists and health policy makers [12].

Numerous reports from Western countries have described an increase in AP use, especially SGA, over recent years [1, 13–17]. These studies differ in terms of studied time period, age groups and other methodological features, thus hampering comparability. While there are some multinational studies comparing antidepressant or ADHD medication use in children and adolescents [18–20], updating patterns of AP use across countries and regions is warranted.

The objective of this study is therefore to determine recent trends in AP use from 2005/2006 through 2012 in 0- to 19 year-olds from five Western countries.

## Methods

### Data sources

#### Denmark

We employed data from the Danish Registry of Medicinal Products Statistics (RMPS). The RMPS is a national prescription database, which encompasses all outpatient pharmacy-dispensed prescription medications in Denmark (5.53 million inhabitants). Each prescription record contains detailed information on the drug dispensed (incl. ATC code). Any drug utilisation prevalence can be calculated using an estimation of the underlying population as denominator.

#### Germany

To perform this study, claims data of the single largest German health insurance company, the BARMER GEK (about 9.1 million insurees, representing more than 10% of the German population) was used. Each prescription record contains detailed information on the prescribed drug, including ATC code. In relation to the complete German population, the BARMER GEK has a slightly higher proportion of female insurees, but there are no differences in terms of socioeconomic status (as measured by education level) [21]. The German data of this study have been published before in a German publication [16].

#### The Netherlands

The data used for this study are pharmacy dispensing data extracted from the IADB.nl database [22]. The IADB.nl database contains all prescription drug dispensing data since 1994 from about 60 community pharmacies. The corresponding population consists of about 600,000 persons from the North East Netherlands. In the Netherlands, patients are generally registered at one pharmacy, and there is an exchange of dispensing data between pharmacies. As a result, a single pharmacy can provide a complete listing of each registered subject’s prescribed drugs history, with the exception of over-the-counter drugs and in-hospital prescriptions. The IADB.nl database population is representative for the whole Dutch population [22].

#### United Kingdom

We used primary care prescribing data from The Health Improvement Network (THIN) primary care database. In the UK National Health Service, primary care doctors (GP’s) are the gatekeepers of referral to both secondary and tertiary care. Children, including those with severe forms of mental disorders, are either not referred for assessment to specialist services or followed up in primary care. THIN holds information on prescriptions issued in general practices (GPs) in all four UK nations. The database covers approximately 6% of the UK population and is broadly representative of the UK population in terms of demographics and consultation behaviour [23]. In this study, we only included practices that had achieved good quality data recording in terms of patient mortality, and average number of records per patient per year [24, 25]. In total, we included 552 practices that contributed data between 2005 and 2012. Overall, prescriptions recorded in THIN reflect redeemed prescriptions, with an average redemption rate of 98.5% in 2008. However, the redemption rate is slightly lower for AP prescriptions at 85.1% in 2008 [26].

#### United States

We used computerized Medicaid administrative claims for the calendar years 2006 through 2012 from a narrowly-defined population of youth (0–19 years) in a mid-Atlantic state enrolled in Children’s Health Insurance Program (CHIP). These children and adolescents are eligible for Medicaid coverage due to family income (upper limit: three times the federal poverty level [27]. The cohort consisted of over 131,000 youth in 2006 and of over 105,000 youth in 2012. Youth who were on Medicaid due to (1) disability; (2) foster care status or (3) family income below poverty level were excluded. Thus the population was similar to privately-insured youth in the US in terms of general health status, age distribution, race and family composition, with moderately lower parental education, employment, and income [28]. Each individual was assigned an encrypted identification number, which was then used to link the enrollment data files to prescription drug claim files.

### Study variables and statistical analysis

Antipsychotics were defined as: all substances designated as class N05A (except Lithium) by the Anatomical Therapeutic Chemical (ATC) Code [29]. Of all AP the following drugs were considered second generation antipsychotics: Amisulpride, aripiprazole, asenapine, clozapine, iloperidone, lurasidone, olanzapine, paliperidone, quetiapine, risperidone, sertindole, sulpiride, ziprasidone and zotepine. The remaining antipsychotic drugs were considered first generation (e.g. chlorprotixene, chlorpromazine, haloperidol and pipamperone).

Annual AP use prevalence was defined as the percentage of youth (0–19 years at the time of prescription) with one or more AP dispensings or prescriptions among continuously enrolled youths in a given calendar year in the 2005/6–2012 period. Rates were not adjusted for age - or sex composition across the cohorts. Relative differences between years were calculated as the difference in prevalence, divided by the prevalence in the first year. The data were stratified by age groups (0–4, 5–9, 10–14, 15–19 years) and gender. The 95% confidence interval for the prevalence rates was calculated with the score method, with continuity correction for small proportions [30]. Differences were considered significant at p < .05.

## Results

### Trends in total use by country and according to age group

**Fig. 1:**
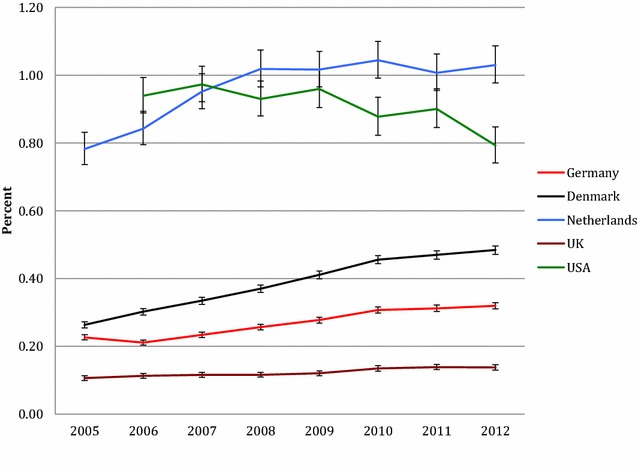
Annual percent prevalence of antipsychotic drug use in children and adolescents (0–19 years) in cohorts from five countries, 2005/6–2012 (with 95% confidence intervals)

From 2005/6 to 2012 the annual prevalence for AP use for youth increased in four of the five countries under study (Fig. 1). This increase was as follows: in Denmark 0.26 to 0.48% (83.9% relative increase), in the German cohort 0.23 to 0.32% (40.8% increase), in the Netherlands’ cohort (0.78 to 1.03% (31.7% increase), and in the UK cohort 0.11 to 0.14% (29.3% increase). A decrease from 0.94 to 0.79% was observed in the US cohort (− 15.6%).

When comparing the prevalence of AP use between countries’ cohorts, large differences were observed (Table 1). In 2012, the highest AP use was observed in the Netherlands’ cohort (1360/131,954; 1.03%), which was eight-fold higher than in the country with the lowest prevalence (UK; 0.14%).

**Table 1:** Annual percent prevalence of antipsychotic drug use in cohorts from five countries between 2005/6–2012 among children and adolescents in 4 age group

	2005	2006	2007	2008	2009	2010	2011	2012	Difference 2005–2012
Denmark									
0–4 years	0.00 [0.00–0.01]	0.00 [0.00–0.01]	0.00 [0.00–0.01]	0.00 [0.00–0.01]	0.00 [0.00–0.01]	0.00 [0.00–0.01]	0.00 [0.00–0.01]	0.00 [0.00–0.00]	N/A
5–9 years	0.07 [0.06–0.08]	0.08 [0.07–0.09]	0.09 [0.08–0.10]	0.10 [0.09–0.11]	0.12 [0.11–0.13]	0.12 [0.11–0.14]	0.11 [0.10–0.13]	0.10 [0.09–0.12]	44.9%
10–14 years	0.26 [0.24–0.28]	0.27 [0.26–0.29]	0.33 [0.31–0.35]	0.34 [0.32–0.37]	0.39 [0.36–0.41]	0.40 [0.38–0.43]	0.40 [0.38–0.42]	0.42 [0.39–0.44]	61.5%
15–19 years	0.77 [0.73–0.80]	0.88 [0.85–0.92]	0.94 [0.90–0.97]	1.03 [0.99–1.06]	1.11 [1.08–1.15]	1.24 [1.21–1.28]	1.30 [1.26–1.34]	1.33 [1.29–1.37]	74.3%
Total	0.26 [0.25–0.27]	0.30 [0.29–0.31]	0.33 [0.32–0.35]	0.37 [0.36–0.38]	0.41 [0.40–0.42]	0.46 [0.44–0.47]	0.47 [0.46–0.48]	0.48 [0.47–0.50]	83.9%
Germany									
0–4 years	0.15 [0.14–0.16]	0.04 [0.03–0.05]	0.02 [0.02–0.03]	0.02 [0.02–0.03]	0.02 [0.02–0.03]	0.02 [0.01–0.02]	0.02 [0.01–0.02]	0.01 [0.01–0.02]	N/A
5–9 years	0.13 [0.12–0.15]	0.13 [0.12–0.14]	0.15 [0.14–0.17]	0.17 [0.15–0.18]	0.18 [0.16–0.19]	0.17 [0.16–0.19]	0.17 [0.16–0.18]	0.17 [0.16–0.18]	25.7%
10–14 years	0.24 [0.23–0.26]	0.27 [0.25–0.28]	0.31 [0.29–0.33]	0.34 [0.32–0.36]	0.37 [0.35–0.39]	0.42 [0.40–0.44]	0.42 [0.41–0.45]	0.43 [0.41–0.45]	76.8%
15–19 years	0.34 [0.33–0.36]	0.34 [0.33–0.36]	0.37 [0.35–0.39]	0.41 [0.39–0.43]	0.44 [0.42–0.46]	0.51 [0.49–0.54]	0.51 [0.52–0.56]	0.54 [0.52–0.56]	57.4%
Total	0.23 [0.22–0.23]	0.21 [0.20–0.22]	0.23 [0.23–0.24]	0.26 [0.25–0.26]	0.28 [0.27–0.29]	0.31 [0.30–0.32]	0.31 [0.31–0.33]	0.32 [0.31–0.33]	40.8%
Netherlands									
0–4 years	0.12 [0.09–0.17]	0.08 [0.06–0.12]	0.09 [0.07–0.13]	0.09 [0.07–0.13]	0.06 [0.04–0.10]	0.09 [0.06–0.13]	0.06 [0.04–0.09]	0.07 [0.05–0.11]	N/A
5–9 years	0.80 [0.71–0.91]	0.87 [0.77–0.98]	1.01 [0.91–1.12]	0.95 [0.85–1.06]	0.97 [0.87–1.08]	0.96 [0.86–1.07]	0.86 [0.77–0.97]	0.84 [0.75–0.95]	5.3%
10–14 years	1.18 [1.06–1.30]	1.32 [1.20–1.45]	1.56 [1.43–1.70]	1.65 [1.51–1.79]	1.68 [1.55–1.83]	1.69 [1.55–1.83]	1.67 [1.53–1.81]	1.59 [1.47–1.73]	35.5%
15–19 years	1.04 [0.94–1.16]	1.12 [1.02–1.24]	1.15 [1.04–1.26]	1.35 [1.24–1.47]	1.44 [1.33–1.57]	1.37 [1.26–1.49]	1.34 [1.23–1.47]	1.47 [1.35–1.60]	40.8%
Total	0.78 [0.74–0.83]	0.84 [0.80–0.89]	0.95 [0.90–1.01]	1.02 [0.97–1.07]	1.02 [0.97–1.07]	1.04 [0.99–1.10]	1.01 [0.96–1.06]	1.03 [0.98–1.09]	31.7%
UK									
0–4 years	0.00 [0.00–0.01]	0.00 [0.00–0.01]	0.00 [0.00–0.00]	0.00 [0.00–0.00]	0.00 [0.00–0.00]	0.00 [0.00–0.01]	0.00 [0.00–0.00]	0.00 [0.00–0.01]	N/A
5–9 years	0.03 [0.03–0.04]	0.03 [0.03–0.04]	0.04 [0.03–0.05]	0.04 [0.03–0.05]	0.04 [0.03–0.05]	0.05 [0.04–0.06]	0.04 [0.03–0.05]	0.03 [0.02–0.04]	− 16.7%
10–14 years	0.12 [0.11–0.14]	0.13 [0.12–0.15]	0.13 [0.12–0.14]	0.14 [0.12–0.15]	0.14 [0.13–0.16]	0.14 [0.13–0.16]	0.15 [0.13–0.16]	0.16 [0.14–0.17]	27.5%
15–19 years	0.25 [0.23–0.28]	0.27 [0.25–0.29]	0.28 [0.26–0.30]	0.26 [0.24–0.28]	0.26 [0.25–0.29]	0.31 [0.29–0.33]	0.33 [0.31–0.35]	0.31 [0.28–0.33]	20.5%
Total	0.11 [0.10–0.11]	0.11 [0.11–0.12]	0.12 [0.11–0.12]	0.12 [0.11–0.12]	0.12 [0.11–0.13]	0.13 [0.13–0.14]	0.14 [0.13–0.15]	0.14 [0.13–0.15]	29.3%
USA									
0–4 years	N/A	0.16 [0.13–0.19]	0.12 [0.10–0.15]	0.10 [0.08–0.13]	0.07 [0.05–0.09]	0.05 [0.03–0.07]	0.04 [0.03–0.07]	0.02 [0.01–0.04]	N/A
5–9 years	N/A	1.31 [1.18–1.47]	1.39 [1.25–1.54]	1.17 [1.04–1.31]	1.04 [0.92–1.18]	0.82 [0.71–0.94]	0.69 [0.59–0.81]	0.56 [0.47–0.66]	− 57.5%
10–14 years	N/A	2.53 [2.33–2.75]	2.59 [2.39–2.82]	2.50 [2.29–2.72]	2.50 [2.29–2.73]	2.23 [2.03–2.44]	2.31 [2.11–2.53]	1.91 [1.73–2.10]	− 24.6%
15–19 years	N/A	2.41 [2.14–2.71]	2.75 [2.47–3.06]	2.87 [2.59–3.19]	3.07 [2.77–3.41]	2.80 [2.50–3.13]	2.69 [2.41–3.01]	2.53 [2.26–2.83]	5.0%
Total	N/A	0.94 [0.89–0.99]	0.97 [0.92–1.03]	0.93 [0.88–0.98]	0.96 [0.91–1.02]	0.88 [0.82–0.94]	0.90 [0.85–0.96]	0.79 [0.74–0.85]	− 15.6%

Numbers in brackets = 95% confidence interval

For the USA, only data from 2006 to 2012 were available

With the exception of the Netherlands’ cohort, AP use was higher in older age cohorts, with 15–19 year-olds showing the highest prevalence (2012: Denmark cohort 1.33%, German cohort 0.54%, Netherlands’ cohort 1.47%, UK cohort 0.31%, US 2.53%). Only in the Netherlands’ cohort AP use prevalence was highest in 10–14 year olds (2012: 1.59%). For 0–4 year olds, after 2008 AP use remained lower than 1 per 1000 in all cohorts.

### Trends in AP use by gender

In all studied cohorts, the prevalence of AP use was higher in boys than in girls (Table 2). In 2012, the male/female ratio ranged from an almost threefold higher use by boys in the Netherlands’ data (2.87) to 1.38 in Denmark.

**Table 2:** Percent prevalence of antipsychotic drug use in 2005/6 and 2012 in 0–19 year-olds in cohorts from 5 countries, divided by gender

	2005 (USA:2006)	M/F ratio	2012	M/F ratio
Denmark				
F	0.22 [0.21–0.23]	1.39	0.40 [0.39–0.42]	1.38
M	0.31 [0.29–0.32]		0.56 [0.54–0.58]	
Germany				
F	0.16 [0.15–0.17]	1.85	0.19 [0.18–0.20]	2.28
M	0.29 [0.28–0.30]		0.44 [0.43–0.46]	
Netherlands				
F	0.37 [0.33–0.42]	3.18	0.51 [0.46–0.57]	2.87
M	1.19 [1.11–1.27]		1.54 [1.45–1.63]	
United Kingdom				
F	0.07 [0.06–0.08]	2.15	0.09 [0.08–0.10]	1.88
M	0.14 [0.13–0.16]		0.18 [0.17–0.19]	
USA (2006)^a^				
F	0.55 [0.50–0.61]	2.39	0.52 [0.46–0.59]	1.95
M	1.32 [1.24–1.40]		1.05 [0.97–1.14]	

Numbers in brackets = 95% confidence interval

For the USA, only data from 2006 to 2012 were available

*M* male; *F* Female

^a^Based on 2006

Across countries, AP use in girls was at or below 0.5% in contrast to AP use in boys that peaked at 1.54% in the Netherlands’ data and 1.05% in the US data. From 2005/6 to 2012 use in boys increased relatively more than in girls in the German cohort, while the opposite was observed in the Netherlands’ and in the UK cohort. In the US data, use in boys decreased more than in girls (− 19.9% vs. − 5.3%). In Denmark, the increase in boys and girls was comparable.

### Patterns in FGA use vs. SGA use by country

**Fig. 2:**
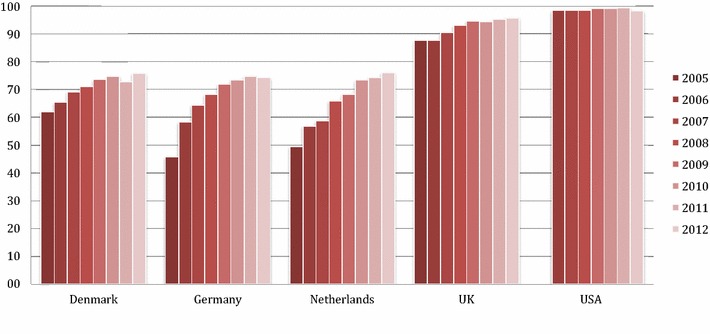
Second generation antipsychotic (SGA) use as a percentage of total antipsychotic use for children and adolescents in cohorts from five countries, 2005/6–2012

In all cohorts except the US cohort the proportion of SGA relative to FGA prescriptions increased (Fig. 2). In the US regional cohort, SGA were almost the only class of drugs used, both in 2006 (98.5% of all prescriptions) and in 2012 (98.3%). In 2005/6 and 2012, risperidone was the most frequently used AP in all countries’ cohorts, with the exception of Denmark, where in 2012 quetiapine ranked first. Use of aripiprazole, a relatively new drug that was approved by the FDA for irritability in autistic children in 2009, increased clearly: While in 2005/6 aripiprazole was only in Denmark and the US data among the top-5 prescribed AP, in 2012 it was in all countries among the five most frequently used AP (Table 3).

**Table 3:** The five most commonly used antipsychotic drugs for children and adolescents in cohorts from five countries, 2005/6 vs 2012

Rank	Denmark				Germany				Netherlands				UK				USA			
	2005	%	2012	%	2005	%	2012	%	2005	%	2012	%	2005	%	2012	%	2006¶	%	2012	%
1	RIS	31.9	QUE	24.1	RIS	30.6	RIS	49.6	RIS	57.8	RIS	51.7	RIS	58.2	RIS	53.8	RIS	57.1	RIS	53.1
2	CHP	24.0	RIS	22.0	PIP	20.4	PIP	16.5	PIP	21.4	QUE	14.4	OLA	14.3	ARI	14.1	ARI	30.2	ARI	31.4
3	OLA	9.8	CHP	21.9	TIA	11.9	QUE	9.5	QUE	6.2	PIP	11.7	HAL	5.4	QUE	14.1	QUE	17.9	QUE	16.9
4	QUE	9.1	ARI	19.0	PMZ	6.7	TIA	6.0	OLA	4.9	ARI	11.0	CPZ	5.3	OLA	11.7	OLA	8.1	ZIP	5.5
5	ARI	4.2	OLA	7.0	OLA	5.8	ARI	4.5	PMZ	3.4	OLA	6.0	QUE	3.9	HAL	1.8	ZIP	4.7	OLA	4.3

For the USA, only data from 2006 to 2012 were available

*ARI* aripiprazole, *CHP* chlorprotixene, *CPZ* chlorpromazine, *HAL* haloperidol, *OLA* olanzapine, *PIP* pipamperone, *PMZ* promazine, *QUE* quetiapine, *RIS* risperidone, *TIA* tiapride, *ZIP* ziprasidone

## Discussion

We observed large differences between samples from 5 countries in the prevalence of AP use, with AP use being highest in the US cohort and lowest in the UK cohort. Since 2007, AP use in the Netherlands’ cohort has surpassed use in the US cohort. Also time trends varied significantly: In the Netherlands’ data, AP use stabilized from 2008 to 2012. In the US cohort, the prevalence of AP use stabilized and decreased towards 2012. All other countries showed a trend for increased use. In most countries’ data, AP use was greatest in 15–19 year-olds. We observed a strong and in most countries increasing preference for SGA, relative to FGA.

There are several possible explanations for the differences in AP use in youth cohorts from different countries: The attitude of prescribers towards psychotropic drugs and antipsychotic drugs and differences in health systems can be a factor that influences AP prescription rates [31]. For example: the attitude of physicians that SGA should be used to treat aggressive behavior can contribute to higher AP prescription rates [32] and the acceptance of psychiatric medication for children by the general public may be a factor [33]. Several studies indicate a broadening of indications, for example in ADHD and other disruptive behaviour disorders [13, 16, 34, 35].

Higher use of AP drugs can be associated with a stronger representation of medical disciplines in the care for youth with behavioral and psychiatric disorders or with an increasing use of mental health care [36]. Gaps in the mental health care system, e.g. lack of social care for the afore-mentioned patient group, may also lead to higher AP prescriptions [37]. It has been demonstrated that longer duration of treatment—and not only more new users—is a relevant factor in the increase in prevalence [14, 38, 39]. The decrease in use in the US confirms recent findings from the US [35] and could be influenced by measures to constrain AP use in youth. For example, recommendations for a more rigorous monitoring of side effects of AP, e.g.: [40, 41] have appeared. In the US, awareness programs targeting clinicians and the public were developed [42] and a system for prior authorization of antipsychotic prescribing for Medicaid insured youth [43] is implemented in 31 states.

We cannot fully explain the higher AP use in the Netherlands (which parallels the Netherlands position in international ADHD medication use [20]) despite the fact that regulatory approval is harmonized across European countries. In the Netherlands, treatment with AP has been included in some guidance statements, but not as a first line treatment option [44]. This finding may reflect a period of emphasis on the biomedical model in Dutch Child and Adolescent Mental Health care. However, the strongest increase in the use of antipsychotics in youth predates the current period under study and unfolded in the period 1995–2005 [14]. It will be worthwhile to observe trends in the Netherlands from 2015 onwards, since important changes have been implemented since 2015 in the position of Child and Adolescent Mental Health care [45], with as one of the objectives a reduction in the use of psychopharmacological drugs in children.

In contrast, the low prescription rates found in the UK cohort may be related to the nature of the UK data, covering only prescriptions issued in primary care. So prescriptions by specialists are not taken into account. Another reason may be that the NICE guideline for ADHD [46] advices against use of antipsychotics in ADHD and the NICE guideline for antisocial behavior and conduct disorders [47] advices against medication as routine management for children with this condition—which stands in contrast to some other countries’ guidelines.

The greatest AP use in 15–19 year-olds in 4 of the 5 countries replicates findings by other authors where AP use increased towards early adolescence [13]. This is an age-group where behavioral problems tend to peak [48] and where severe mood disorders and psychotic disorders emerge. Another factor may be reluctance in prescribers towards prescribing for younger patients. The highest use in 10–14 year-olds that was found in the Netherlands may be explained by more use in behavioral disorders and by less reluctance towards prescribing in younger patients.

One explanation for the strong trend towards the use of SGA—which constitutes an exceptional growth in comparison to older studies (in 2000, in Germany only 5% of AP were SGA, [49])—may be that the literature about AP in youth is dominated by SGA focused papers, although the actual evidence base for efficacy is weak for most indications [50]. This may possibly an effect of more investment in the development and registration process of newer drugs. Previously, SGA were also considered more safe due to a smaller risk for extrapyramidal side effects [51] and tardive dyskinesia [52]. The insight that SGA are associated with different, but not necessarily smaller risks than FGA [53] is of more recent date since most reports about metabolic and endocrinological side effects have appeared in the last decade [40, 54–58].

### Limitations, and implications of this study

This study is one of the first to describe use of antipsychotics in youth cohorts from different countries. The diversity of the underlying databases is a limitation as the underlying populations differ and this will certainly influence the rates that we found: The Danish cohort is nationwide, the US cohort comprises CHIP insured patients from one state, the Netherlands cohort covers a region of the country, the German cohort comprises patients from one large insurance company, while the UK cohort covers prescriptions from primary care. So, between-country comparisons should be made with caution. We were not able to control for co-medication, prescribing physician specialty (GPs vs. specialists) or socio-economic status, factors which influence AP use [51, 59]. Our data sources lack information that could improve the perspective on AP use, such as underlying indication, ethnic background, foster care status, duration of pharmacotherapy, adherence, symptom severity and symptom duration. We did not consider medication for hospitalized children. But the number of hospitalized youth may be small, compared to outpatients [60], and usually medication is continued in the outpatient setting after discharge from hospital.

In this vein, future studies will benefit from the use of harmonized databases, information about diagnosis (e.g. [61]) and use of other treatments, concurrent or sequential, thus giving more insight on indications and unmet needs in care across populations [59]. Data about incidence and duration of AP use is relevant, since longer exposure to the metabolic and endocrinological side effects of AP poses higher risks for health.

The implications of this study are that guidelines and practice parameters for AP use drugs need closer scrutiny. For those drugs where efficacy has been demonstrated in RCTs of limited duration, there is a pressing need for longer lasting observational and discontinuation studies to determine the risks and benefits of long-term use [62–64]. Close monitoring of use of psychopharmacological agents over time and across countries may sensitize to national discrepancies in mental health care, differences in use of psychopharmacological treatment and populations with special needs or risks. For this purpose, a fixed multinational set of databases, gauged against each other, is an essential tool.

## Abbreviations

AP: antipsychotics
FGA: first generation antipsychotic drugs
SGA: second generation antipsychotic drugs
UK: United Kingdom
USA/US: United States of America
ARI: aripiprazole
CHP: chlorprotixene
CPZ: chlorpromazine
HAL: haloperidol
OLA: olanzapine
PIP: pipamperone
PMZ: promazine
QUE: quetiapine
RIS: risperidone
TIA: tiapride
ZIP: ziprasidone

## References

[1] Verdoux H, Tournier M, Begaud B (2010). Antipsychotic prescribing trends: a review of pharmaco-epidemiological studies. Acta Psychiatr Scand.

[2] Malone RP, Sheikh R, Zito JM (1999). Novel antipsychotic medications in the treatment of children and adolescents. Psychiatr Serv.

[3] Glazer WM (2000). Extrapyramidal side effects, tardive dyskinesia, and the concept of atypicality. J Clin Psychiatry.

[4] Stafford MR, Mayo-Wilson E, Loucas CE, James A, Hollis C, Birchwood M, Kendall T (2015). Efficacy and safety of pharmacological and psychological interventions for the treatment of psychosis and schizophrenia in children, adolescents and young adults: a systematic review and meta-analysis. PLoS ONE.

[5] Liu HY, Potter MP, Woodworth KY, Yorks DM, Petty CR, Wozniak JR, Faraone SV, Biederman J (2011). Pharmacologic treatments for pediatric bipolar disorder: a review and meta-analysis. J Am Acad Child Adolesc Psychiatry.

[6] Troost PW, Lahuis BE, Steenhuis MP, Ketelaars CE, Buitelaar JK, van Engeland H, Scahill L, Minderaa RB, Hoekstra PJ (2005). Long-term effects of risperidone in children with autism spectrum disorders: a placebo discontinuation study. J Am Acad Child Adolesc Psychiatry.

[7] Hollis C, Pennant M, Cuenca J, Glazebrook C, Kendall T, Whittington C, Stockton S, Larsson L, Bunton P, Dobson S, Groom M, Hedderly T, Heyman I, Jackson GM, Jackson S, Murphy T, Rickards H, Robertson M, Stern J (2016). Clinical effectiveness and patient perspectives of different treatment strategies for tics in children and adolescents with Tourette syndrome: a systematic review and qualitative analysis. Health Technol Assess.

[8] Pringsheim T, Hirsch L, Gardner D, Gorman DA (2015). The pharmacological management of oppositional behaviour, conduct problems, and aggression in children and adolescents with attention-deficit hyperactivity disorder, oppositional defiant disorder, and conduct disorder: a systematic review and meta-analysis. Part 2: antipsychotics and traditional mood stabilizers. Can J Psychiatry.

[9] Loy JH, Merry SN, Hetrick SE, Stasiak K (2012). Atypical antipsychotics for disruptive behaviour disorders in children and youths. Cochrane Database Syst Rev.

[10] de Bildt A, Mulder EJ, Scheers T, Minderaa RB, Tobi H (2006). Pervasive developmental disorder, behavior problems, and psychotropic drug use in children and adolescents with mental retardation. Pediatrics.

[11] Ben Amor L (2012). Antipsychotics in pediatric and adolescent patients: a review of comparative safety data. J Affect Disord.

[12] Olfson M (2012). Epidemiologic and clinical perspectives on antipsychotic treatment of children and adolescents. Can J Psychiatry.

[13] Olfson M, King M, Schoenbaum M (2015). Treatment of young people with antipsychotic medications in the United States. JAMA Psychiatry.

[14] Kalverdijk LJ, Tobi H, van den Berg PB, Buiskool J, Wagenaar L, Minderaa RB, de Jong-van den Berg LT (2008). Use of antipsychotic drugs among Dutch youths between 1997 and 2005. Psychiatr Serv.

[15] Patten SB, Waheed W, Bresee L (2012). A review of pharmacoepidemiologic studies of antipsychotic use in children and adolescents. Can J Psychiatry.

[16] Bachmann CJ, Lempp T, Glaeske G, Hoffmann F (2014). Antipsychotic prescription in children and adolescents: an analysis of data from a German statutory health insurance company from 2005 to 2012. Dtsch Arztebl Int.

[17] Burcu M, Zito JM, Ibe A, Safer DJ (2014). Atypical antipsychotic use among medicaid-insured children and adolescents: duration, safety, and monitoring implications. J Child Adolesc Psychopharmacol.

[18] Zito JM, Tobi H, de Jong-van den Berg LT, Fegert JM, Safer DJ, Janhsen K, Hansen DG, Gardner JF, Glaeske G (2006). Antidepressant prevalence for youths: a multi-national comparison. Pharmacoepidemiol Drug Saf.

[19] Bachmann CJ, Aagaard L, Burcu M, Glaeske G, Kalverdijk LJ, Petersen I, Schuiling-Veninga CC, Wijlaars L, Zito JM, Hoffmann F (2016). Trends and patterns of antidepressant use in children and adolescents from five western countries, 2005–2012. Eur Neuropsychopharmacol.

[20] Bachmann. CJ, Wijlaars L, Kalverdijk LJ, Burcu M, Glaeske G, Petersen I, Schuiling-Veninga CM, Hoffmann F, Zito JM. Trends in ADHD medication use in children and adolescents in five Western countries, 2005–2012. 2016 **(Under review)**.

[21] Hoffmann F, Bachmann CJ (2014). Differences in sociodemographic characteristics, health, and health service use of children and adolescents according to their health insurance funds. Bundesgesundheitsblatt Gesundheitsforschung Gesundheitsschutz.

[22] Visser ST, Schuiling-Veninga CC, Bos JH, de Jong-van den Berg LT, Postma MJ (2013). The population-based prescription database IADB.nl: its development, usefulness in outcomes research and challenges. Expert Rev Pharmacoecon Outcomes Res.

[23] Blak BT, Thompson M, Dattani H, Bourke A (2011). Generalisability of The Health Improvement Network (THIN) database: demographics, chronic disease prevalence and mortality rates. Inform Prim Care.

[24] Horsfall L, Walters K, Petersen I (2013). Identifying periods of acceptable computer usage in primary care research databases. Pharmacoepidemiol Drug Saf.

[25] Maguire A, Blak BT, Thompson M (2009). The importance of defining periods of complete mortality reporting for research using automated data from primary care. Pharmacoepidemiol Drug Saf.

[26] Health and social care information centre. The prescribing compliance a review of the proportion of prescriptions dispensed. http://www.hscic.gov.uk/home. 2011. Accessed 02 Jan 2017.

[27] http://kff.org/health-reform/state-indicator/medicaid-and-chip-income-eligibility-limits-for-children-as-a-percent-of-the-federal-poverty-level/. Accessed 02 Jan 2017.

[28] Byck GR (2000). A comparison of the socioeconomic and health status characteristics of uninsured, state children’s health insurance program-eligible children in the united states with those of other groups of insured children: implications for policy. Pediatrics.

[29] World Health Organization: (2016) ATC/DDD index. http://www.whocc.no/atc_ddd_index/. Accessed 05 Jan 2017.

[30] Tobi H, van den Berg PB, de Jong-van den Berg LT (2005). Small proportions: what to report for confidence intervals?. Pharmacoepidemiol Drug Saf.

[31] Schomerus G, Matschinger H, Baumeister SE, Mojtabai R, Angermeyer MC (2014). Public attitudes towards psychiatric medication: a comparison between United States and Germany. World Psychiatry.

[32] Rodday AM, Parsons SK, Correll CU, Robb AS, Zima BT, Saunders TS, Leslie LK (2014). Child and adolescent psychiatrists’ attitudes and practices prescribing second generation antipsychotics. J Child Adolesc Psychopharmacol.

[33] McLeod JD, Pescosolido BA, Takeuchi DT, White TF (2004). Public attitudes toward the use of psychiatric medications for children. J Health Soc Behav.

[34] Penfold RB, Stewart C, Hunkeler EM, Madden JM, Cummings JR, Owen-Smith AA, Rossom RC, Lu CY, Lynch FL, Waitzfelder BE, Coleman KJ, Ahmedani BK, Beck AL, Zeber JE, Simon GE (2013). Use of antipsychotic medications in pediatric populations: what do the data say?. Curr Psychiatry Rep.

[35] Crystal S, Mackie T, Fenton MC, Amin S, Neese-Todd S, Olfson M, Bilder S (2016). Rapid growth of antipsychotic Prescriptions for children who Are publicly insured has ceased but concerns remain. Health Aff (Millwood).

[36] Steinhausen HC (2015). Recent international trends in psychotropic medication prescriptions for children and adolescents. Eur Child Adolesc Psychiatry.

[37] Murphy AL, Gardner DM, Kisely S, Cooke CA, Kutcher SP, Hughes J (2015). System struggles and substitutes: a qualitative study of general practitioner and psychiatrist experiences of prescribing antipsychotics to children and adolescents. Clin Child Psychol Psychiatry.

[38] Abbas S, Ihle P, Adler JB, Engel S, Gunster C, Linder R, Lehmkuhl G, Schubert I (2016). Psychopharmacological Prescriptions in Children and Adolescents in Germany. Dtsch Arztebl Int.

[39] Rani F, Murray ML, Byrne PJ, Wong IC (2008). Epidemiologic features of antipsychotic prescribing to children and adolescents in primary care in the United Kingdom. Pediatrics.

[40] Correll CU, Carlson HE (2006). Endocrine and metabolic adverse effects of psychotropic medications in children and adolescents. J Am Acad Child Adolesc Psychiatry.

[41] Cahn W, Ramlal D, Bruggeman R, de Haan L, Scheepers FE, van Soest MM, Assies J, Slooff CJ (2008). Prevention and treatment of somatic complications arising from the use of antipsychotics. Tijdschr Psychiatr.

[42] ABIM Foundation American Psychiatric Association (2015). Five things physicians and patients should question. http://www.choosingwisely.org/clinicianlists/american-psychiatric-association-antipsychotics-in-children-or-adolescents/. Accessed 27 Jan 2017.

[43] Schmid I, Burcu M, Zito JM (2015). Medicaid prior authorization policies for pediatric use of antipsychotic medications. JAMA.

[44] Kenniscentrum (2017) Landelijk Kenniscentrum Kinder- en Jeugpsychiatrie. http://www.kenniscentrum-kjp.nl/en/home. Accessed 05 Jan 2017.

[45] Hilverdink P, Daamen W, Vink C. Children and youth support and care in the Netherlands. Neth Youth Inst. (www.nji.nl/english); 2015:8.

[46] NICE. Attention deficit hyperactivity disorder: diagnosis and management. Clinical guideline [CG72]. 2008. https://www.nice.org.uk/guidance/cg72. Accessed 01 Aug 2017.

[47] NICE. Antisocial behaviour and conduct disorders in children and young people: recognition and treatment. [CG158]. 2013. https://www.nice.org.uk/guidance/cg158. Accessed 01 Sept 2017.

[48] Moffitt TE (1993). Adolescence-limited and life-course-persistent antisocial behavior: a developmental taxonomy. Psychol Rev.

[49] Zito JM, Safer DJ, de Jong-van den Berg LT, Janhsen K, Fegert JM, Gardner JF, Glaeske G, Valluri SC (2008). A three-country comparison of psychotropic medication prevalence in youth. Child Adolesc Psychiatry Ment Health.

[50] Pringsheim T, Gorman D (2012). Second-generation antipsychotics for the treatment of disruptive behaviour disorders in children: a systematic review. Can J Psychiatry.

[51] Correll CU (2008). Antipsychotic use in children and adolescents: minimizing adverse effects to maximize outcomes. J Am Acad Child Adolesc Psychiatry.

[52] Correll CU, Leucht S, Kane JM (2004). Lower risk for tardive dyskinesia associated with second-generation antipsychotics: a systematic review of 1-year studies. Am J Psychiatry.

[53] Leucht S, Corves C, Arbter D, Engel RR, Li C, Davis JM (2009). Second-generation versus first-generation antipsychotic drugs for schizophrenia: a meta-analysis. Lancet.

[54] Correll CU, Lencz T, Malhotra AK (2011). Antipsychotic drugs and obesity. Trends Mol Med.

[55] Andrade SE, Lo JC, Roblin D, Fouayzi H, Connor DF, Penfold RB, Chandra M, Reed G, Gurwitz JH (2011). Antipsychotic medication use among children and risk of diabetes mellitus. Pediatrics.

[56] Bobo WV, Cooper WO, Stein CM, Olfson M, Graham D, Daugherty J, Fuchs DC, Ray WA (2013). Antipsychotics and the risk of type 2 diabetes mellitus in children and youth. JAMA Psychiatry.

[57] Correll CU, Manu P, Olshanskiy V, Napolitano B, Kane JM, Malhotra AK (2009). Cardiometabolic risk of second-generation antipsychotic medications during first-time use in children and adolescents. JAMA.

[58] Roke Y, Buitelaar JK, Boot AM, Tenback D, van Harten PN (2012). Risk of hyperprolactinemia and sexual side effects in males 10–20 years old diagnosed with autism spectrum disorders or disruptive behavior disorder and treated with risperidone. J Child Adolesc Psychopharmacol.

[59] Sikirica V, Pliszka SR, Betts KA, Hodgkins P, Samuelson T, Xie J, Erder H, Dammerman R, Robertson B, Wu EQ (2012). Comparative treatment patterns, resource utilization, and costs in stimulant-treated children with ADHD who require subsequent pharmacotherapy with atypical antipsychotics versus non-antipsychotics. J Manag Care Pharm.

[60] Graaf Md, Schouten R, Konijn C. De Nederlandse jeugdzorg in cijfers 1998–2002. NIZW Jeugd. 2005.

[61] Nesvag R, Hartz I, Bramness JG, Hjellvik V, Handal M, Skurtveit S (2016). Mental disorder diagnoses among children and adolescents who use antipsychotic drugs. Eur Neuropsychopharmacol.

[62] Rani FA, Byrne PJ, Murray ML, Carter P, Wong IC (2009). Paediatric atypical antipsychotic monitoring safety (PAMS) study: pilot study in children and adolescents in secondary- and tertiary-care settings. Drug Saf.

[63] Glennon J, Purper-Ouakil D, Bakker M, Zuddas A, Hoekstra P, Schulze U, Castro-Fornieles J, Santosh PJ, Arango C, Kolch M, Coghill D, Flamarique I, Penzol MJ, Wan M, Murray M, Wong IC, Danckaerts M, Bonnot O, Falissard B, Masi G, Fegert JM, Vicari S, Carucci S, Dittmann RW, Buitelaar JK, PERS Consortium (2014). Paediatric European Risperidone Studies (PERS): context, rationale, objectives, strategy, and challenges. Eur Child Adolesc Psychiatry.

[64] Persico AM, Arango C, Buitelaar JK, Correll CU, Glennon JC, Hoekstra PJ, Moreno C, Vitiello B, Vorstman J, Zuddas A, European Child and Adolescent Clinical Psychopharmacology Network (2015). Unmet needs in paediatric psychopharmacology: present scenario and future perspectives. Eur Neuropsychopharmacol.

